# Exercise and Bone Mineral Density in Premenopausal Women: A Meta-Analysis of Randomized Controlled Trials

**DOI:** 10.1155/2013/741639

**Published:** 2013-01-17

**Authors:** George A. Kelley, Kristi S. Kelley, Wendy M. Kohrt

**Affiliations:** ^1^Department of Community Medicine, West Virginia University, Morgantown, WV 26506, USA; ^2^Meta-Analytic Research Group, Department of Biostatistics, School of Public Health, Robert C. Byrd Health Sciences Center, West Virginia University, P.O. Box 9190, Morgantown, WV 26506-9190, USA; ^3^Division of Geriatric Medicine, University of Colorado Denver, Anschutz Medical Campus, P.O. Box 6511, Mail Stop B179, 12631 East 17th Avenue-L15, Aurora, CO 80045, USA

## Abstract

*Objective*. Examine the effects of exercise on femoral neck (FN) and lumbar spine (LS) bone mineral density (BMD) in premenopausal women. *Methods*. Meta-analysis of randomized controlled exercise trials ≥24 weeks in premenopausal women. Standardized effect sizes (*g*) were calculated for each result and pooled using random-effects models, *Z* score alpha values, 95% confidence intervals (CIs), and number needed to treat (NNT). Heterogeneity was examined using *Q* and *I* ^2^. Moderator and predictor analyses using mixed-effects ANOVA and simple metaregression were conducted. Statistical significance was set at *P* ≤ 0.05. *Results*. Statistically significant improvements were found for both FN (7*g*'s, 466 participants, *g* = 0.342, 95%  CI = 0.132, 0.553, *P* = 0.001, *Q* = 10.8, *P* = 0.22, *I* ^2^ = 25.7%, NNT = 5) and LS (6*g*'s, 402 participants, *g* = 0.201, 95%  CI = 0.009, 0.394, *P* = 0.04, *Q* = 3.3, *P* = 0.65, *I* ^2^ = 0%, NNT = 9) BMD. A trend for greater benefits in FN BMD was observed for studies published in countries other than the United States and for those who participated in home versus facility-based exercise. Statistically significant, or a trend for statistically significant, associations were observed for 7 different moderators and predictors, 6 for FN BMD and 1 for LS BMD. *Conclusions*. Exercise benefits FN and LS BMD in premenopausal women. The observed moderators and predictors deserve further investigation in well-designed randomized controlled trials.

## 1. Introduction

 Bone is a living tissue that undergoes continuous remodeling as a result of bone resorption and formation whereby osteoclasts remove bone and osteoblasts create new bone [1]. A dynamic tissue, bone, adapts to the associated mechanical stresses, such as exercise, that are placed on it [2]. Currently, mechanotransduction is the predominant mechanism through which mechanical stimuli such as exercise are believed to benefit bone [3, 4]. While not entirely understood, this appears to involve the detection of mechanical stimuli by osteocytes and the transduction of this mechanical strain by osteocytes to osteoclasts and osteoblasts where bone resorption and remodeling take place [4, 5], the end result being enhanced bone formation. At the cellular level, exercise may reduce the secretion of sclerostin by the osteocyte, thereby upregulating Wnt signaling and osteoblastogenesis, that is, bone formation [6–8]. To support this contention, both cross-sectional and longitudinal studies have shown that physically active premenopausal women have lower sclerostin levels than those who are sedentary [9, 10]. In a cross-sectional study of 1,235 randomly selected premenopausal women, those who participated in more than 120 minutes of physical activity per week were shown to have serum sclerostin levels that were 36.8% lower than sedentary controls [9]. In a longitudinal follow-up study with 120 of these same women who took part in either an 8-week, 4 days per week, exercise (*n* = 58) or control (*n* = 62) condition, serum sclerostin levels were 33.9% lower in the exercise versus control group [9]. 

 Maintaining optimal bone mineral density (BMD) levels during the premenopausal years is important for reducing the risk of osteoporosis and subsequent fractures during the postmenopausal years, with relative-risk increases ranging from 1.5 to 3.0 [11]. In addition, the prevalence of osteopenia and osteoporosis has been reported to be 15% and 0.6%, respectively, in premenopausal women [12]. Furthermore, it has been estimated that the loss of BMD ranges from 0.25% to 1% per year in premenopausal women [11]. While pharmacologic therapy is usually contraindicated in premenopausal women, reliance on lifestyle factors is almost always recommended [11, 13]. One potentially effective lifestyle approach for achieving this goal is exercise, a low-cost, nonpharmacologic intervention that is available to the vast majority of the population. Unfortunately, previous randomized controlled trials addressing the effects of joint and/or ground reaction force exercise on femoral neck (FN) and lumbar spine (LS) BMD in premenopausal women have led to conflicting and less than overwhelming results, with only 30% and 29% of findings reported as statistically significant at the FN and LS, respectively [14–20]. Using the traditional vote-counting approach [21], one might conclude that exercise does not benefit FN or LS BMD. However, a vote-counting approach based on statistical significance can be extremely misleading since the absence of a statistically significant effect does not mean absence of an effect [21]. In contrast, meta-analysis is a quantitative approach that enables one to go beyond statistical significance and focus on the magnitude of effect [22]. 

 While a number of meta-analyses have been conducted on the effects of exercise on BMD in adults [23–45], none have focused exclusively on FN and/or LS BMD when limited to randomized controlled trials in premenopausal women. However, three meta-analyses have reported subgroup findings when limited to randomized controlled trials [37, 41, 44]. First, Wallace and Cumming reported a statistically significant and positive effect of both impact (1.5%) and nonimpact (1.2%) exercises on LS BMD [44]. A nonsignificant improvement of approximately 0.9% was found at the FN after impact exercise while an insufficient number of studies were available to examine nonimpact exercise [44]. A second meta-analysis that was limited to high-intensity resistance training reported a statistically significant benefit of 0.013 g/cm^2^ for LS BMD and a nonsignificant effect of 0.001 g/cm^2^ for FN BMD [37]. Based on a random-effects model and across all interventions, a third meta-analysis by the same research group reported a statistically significant benefit of 0.007 g/cm^2^ at the LS and 0.012 g/cm^2^ at the FN as a result of different impact modalities [41]. While the results of these meta-analyses are important, none were limited to randomized controlled trials. This is potentially problematic because randomized controlled trials are the only way to control for confounders that are not known or measured as well as the observation that nonrandomized controlled trials tend to overestimate the effects of healthcare interventions [46, 47]. In addition, none of these meta-analyses conducted moderator analyses for other variables when limited to randomized controlled trials [37, 41, 44]. Furthermore, none of the studies [37, 41, 44] provided any quantitative assessment of clinical relevance with respect to the number needed to treat (NNT) [48]. Given the former, the purpose of this study was to use the aggregate data meta-analytic approach to determine the overall effects, as well as potential moderators and predictors, of ground and joint reaction force exercise on FN and LS BMD in premenopausal women. 

## 2. Methods

### 2.1. Study Eligibility Criteria

 Studies were included if they met the following criteria: (1) randomized trials with a comparative control group (for example, nonintervention), (2) premenopausal women, as defined by the authors, (3) participants not engaged in a regular exercise program prior to study enrollment, (4) ground and/or joint reaction force exercise intervention of at least 24 weeks, (5) published and unpublished (master's theses and dissertations) studies since January 1989, and (6) data available for changes in BMD at the FN and/or LS and assessed using dual-energy X-ray absorptiometry (DEXA) or dual-photon absorptiometry (DPA). Any studies not meeting all six criteria were excluded. 

Studies were limited to randomized controlled trials because trials are the only way to control for confounders that are not known or measured as well as the observation that nonrandomized controlled trials tend to overestimate the effects of healthcare interventions [46, 47]. The rationale for limiting studies to those in which the exercise intervention was at least 24 weeks in duration was based on the fact that bone remodeling, a continuous process in which damaged bone is repaired, ion homeostasis is maintained, and bone is reinforced for increased stress, typically takes around 24 weeks [49, 50]. Thus, it is unlikely that any true exercise-induced skeletal changes in BMD would occur prior to this. Because of the site specificity of exercise on BMD [51], resistance training studies were limited to those that included lower body exercise. The year 1989 was chosen as the start date for inclusion since it appeared to be the first time that a randomized controlled trial on exercise and BMD in adult humans was conducted [52].

### 2.2. Data Sources

 Studies were retrieved from a large, previously developed database that included 1055 unique citations (see flow diagram in Supplementary File 1, available online at http://dx.doi.org/10.1155/2013/741639). Citations for the original database were retrieved from (1) six electronic sources (PubMed, Embase, SportDiscus, Cochrane Central Register of Controlled Clinical Trials, CINAHL, Dissertation Abstracts International), (2) cross-referencing from retrieved studies, including previous reviews, and (3) hand searching selected journals. Keywords germane to all searches were “exercise,” “bone,” and “randomized.” In consultation with a Health Sciences librarian at West Virginia University, all searches were conducted by the second author (K. Kelly). The last search was conducted in August of 2011. In accordance with recent guidelines [53], an example of the search strategy used for one of the electronic databases (CINAHL) is shown in Supplementary File 2. Based on previous research suggesting that searching for unpublished data is probably not worth the effort, no attempt was made to retrieve such [54].

### 2.3. Study Selection

 All studies were selected by the first two authors (G. Kelley and K. Kelley), independent of each other. They then reviewed their selections for accuracy and consistency. Discrepancies were resolved by consensus. If consensus could not be reached, the third author (W. Kohrt) was consulted and asked to provide a recommendation. The final list of selected studies was reviewed for thoroughness and completeness by the third author (W. Kohrt), an expert on exercise and BMD. A list of included and excluded studies, including the reasons for exclusion, was stored in version 12 of Reference Manger [55]. 

### 2.4. Data Extraction

 Prior to data extraction, electronic codebooks were developed using Microsoft Excel 2007 [56]. Initial codebooks were developed by the first author (G. Kelley) with input from the second and third authors. Each codebook was then reviewed and tested by all three authors. Codebooks were then revised by the first author (G. Kelley) and reviewed and tested by all authors until final codebooks for data extraction were available after three iterations. The major categories of variables coded included (1) study characteristics (year of publication, risk of bias, etc.), (2) group characteristics (age, height, etc.) and (3) outcome characteristics (changes in FN and LS BMD, secondary outcomes, etc.). Codebooks could hold up to 324 items from each study. 

 The primary outcomes for this study, determined *a priori*, were changes in FN and LS BMD assessed by DEXA or DPA. Secondary outcomes, also established *a priori*, included changes in other BMD sites (whole body, Ward's triangle, intertrochanter, trochanter, total hip, radius, ulna, calcaneus, and os calcis), body weight, body mass index, lean body mass, percent body fat, fat mass, muscular strength (upper and/or lower), muscular power, cardiorespiratory fitness, balance (static and dynamic), calcium intake, vitamin D intake, and fractures. 

 All data were extracted by the first two authors (G. Kelley and K. Kelley), independent of each other. They then met and reviewed every selection for accuracy and consistency. Discrepancies were resolved by consensus. If consensus could not be reached, the third author (W. Kohrt) served as an arbitrator. Trials published as duplicate reports (parallel publications) were only included once, using all associated trial reports to maximally extract trial information, but ensuring that the trial data were not duplicated in the review.

### 2.5. Risk of Bias Assessment

 Risk of bias was assessed using the risk of bias assessment tool from the Cochrane Collaboration [57]. This tool addresses specific domains, namely, sequence generation, allocation concealment, blinding of participants and personnel, blinding of outcome assessment, incomplete outcome data, and selective outcome reporting. Each domain is classified as having either a high, low, or unclear risk of bias [57]. Given the objective nature of BMD assessment, all studies were considered low risk with respect to blinding. For selective outcome reporting, all studies were considered to be at an unclear risk for bias unless a study protocol identification number was provided. If a study protocol identification number was provided, an *a priori* decision was made to locate the project on the respective clinical trials website to see if the number and type of outcomes reported in the study matched the number and type of outcomes reported on the website. Risk of bias was assessed by the first two authors (G. Kelley and K. Kelley). They then met and reviewed every item for agreement. Disagreements were resolved by consensus. 

### 2.6. Statistical Analysis

#### 2.6.1. Calculation of Effect Sizes from Each Study 

The primary outcomes for this study, that is, changes in FN and LS BMD, were calculated using the standardized effect size *g* [58]. The standardized effect size was chosen over the original metric because of the different methods used to report data, for example, absolute versus relative changes in BMD, as well as the potential for excluding eligible studies because of the inability to retrieve necessary data. Each *g* was calculated as follows [58]:

(1) $$\begin{array}{c} g_{i}=\frac{{\overset{-}{X}}_{e}-\;{\overset{-}{X}}_{c}}{\;{\text{SD}}_{\text{pooled}}}, \end{array}$$

where ${\overset{-}{X}}_{e}$ represents the changes score difference in the exercise group, ${\overset{-}{X}}_{c}$ represents the change score difference in the control group, and SD_pooled_ represents the pooled standard deviation from the change score standard deviations of the exercise and control groups. If absolute data were not available, relative (percent change) data were used. 

 For those studies that did not report original metric change score standard deviations, these were calculated from 95% confidence intervals if they were reported. If change score standard deviations and 95% confidence intervals were not available, change score standard deviations for each group (exercise and control) were calculated using the estimation approach of Follmann et al. [59]:

(2) $$\begin{array}{c} \text{SD}=\sqrt{\left({\text{SD}}_{\text{initial}}^{2}+{\text{SD}}_{\text{final}}^{2}\right)-2\left({\text{SD}}_{\text{initial}}*{\text{SD}}_{\text{final}}*{\text{Corr}}_{\text{intial},\text{final}}\right)\;}, \end{array}$$

where SD_pre_^2^ is the square of the standard deviation for the initial score, SD_post_^2^  is the square of the standard deviation for the final score, and Corr_pre,post_ is the correlation between initial and final scores. Based on the association between initial and final scores, the imputed correlation for this study was 0.90. After original metric change score standard deviations were calculated from each study, the pooled standard deviation for *g* was calculated as follows [58]:

(3) $$\begin{array}{c} {\text{SD}}_{\text{pooled}}=\sqrt{\frac{\left(n_{e}-1\right){\text{SD}}_{e}^{2}+\left(n_{c}-1\right){\text{SD}}_{c}^{2}}{n_{e}+n_{c}-2}}, \end{array}$$

where SD_pooled_ is the pooled standard deviation for *g*, *n*_*e*_ is the sample size in the exercise group, *n*_*c*_ is the sample size in the control group, SD_*e*_^2^ is the square of the standard deviation in the exercise group, and SD_*c*_^2^ is the square of the standard deviation in the control group. Each *g* was then corrected for small sample bias by multiplying *g* by a constant [58]:

(4) $$\begin{array}{c} g_{i}^{*}=c_{i}g_{i}, \end{array}$$

where

(5) $$\begin{array}{c} c_{i\;\approx }1-\frac{3}{4\left(n_{e}+\;n_{c}-\;2\right)-1\;\;}. \end{array}$$

The variance for each *g* was then calculated as follows [58]:

(6) $$\begin{array}{c} {\mathrm{Var}}_{g_{i}}=\frac{n_{e}+n_{c}}{n_{e}n_{c}}+\;\frac{g_{i}^{2}}{2\left(n_{e}+n_{c}\right)}, \end{array}$$

where Var⁡_*g*_*i*__ is the variance for *g*, *n*_*e*_ is the sample size in the exercise group, and *n*_*c*_ is the sample size in the control group. For pooling purposes, each *g* was then weighted by the inverse of the variance as follows [58]:

(7) $$\begin{array}{c} w_{i}=\;\frac{1}{{\mathrm{Var}}_{g_{i}}}, \end{array}$$

where *w*_*i*_ represents the weight and Var⁡_*g*_*i*__ is the variance for each *g*.

 Effect sizes for secondary outcomes (whole body BMD, Ward's triangle, intertrochanter, trochanter, total hip, radius, ulna, calcaneus, os calcis, upper and low body muscular strength, muscular power, and static and dynamic balance) were also calculated using *g*. Generally, the magnitude of effect for *g* may be classified as trivial (<0.20), small (≥0.20 to <0.50), medium (≥0.50 to <0.80), or large (≥0.80) [60]. A *g* of 0.30, for example, means that exercise would result in a 0.30 SD benefit over those who did not exercise. The original metric was used to calculate all other secondary outcomes: cardiorespiratory fitness (VO_2 max_ in mL/kg^−1^/min⁡^−1^), body weight (kg), body mass index (kg/m^2^), lean body mass (kg), percent body fat (%), fat mass (kg) calcium intake (mg/day), vitamin D intake (IU), and number of fractures.

#### 2.6.2. Effect Size Pooling

All effect sizes were pooled using a random-effects, method of moments model [61]. This approach weights studies by the inverse of the variance and incorporates heterogeneity into the model [61]. For both primary and secondary outcomes, pooling was limited to those outcomes with at least 3 effect sizes. Multiple groups from the same study were analyzed independently as well as collapsing multiple groups so that only one effect size represented each outcome from each study. A two-tailed *Z* score alpha value of ≤0.05 was considered to be statistically significant while alpha values >0.05 but ≤0.10 were considered as a trend. Precision was determined using two-tailed 95% confidence intervals (CIs). For outcomes with statistically significant results, estimation of treatment effects in a new trial was calculated using 95% prediction intervals (PIs) [62–64]. To enhance clinical relevance, the NNT was also estimated [48]. Analysis of secondary outcomes was considered exploratory because they were not part of the inclusion criteria, and thus, may represent a biased sample. After initial pooling, studies with statistically significant residuals (outliers) were deleted from all further analysis. The alpha value for statistically significant residuals was set at *P* ≤ 0.05. Because of a lack of data (<3 effect sizes), analysis of secondary outcomes was limited to changes in body weight and BMD at Ward's triangle and the trochanteric regions. 

 Statistical heterogeneity of pooled results based on fixed-effects models was examined using the *Q* statistic and *I* ^2^, an extension of *Q* that more accurately reflects statistical heterogeneity [65]. The alpha value for statistical significance for *Q* was set at *P* ≤ 0.10. For *I* ^2^, values of 25% to <50% may be considered small, 50% to <75% medium, and ≥75% large [65]. For this study, *I* ^2^ values >50% were considered as excessive heterogeneity. Potential bias due to small-study effects was examined using the approach of Egger et al. and an alpha value for statistical significance of *P* ≤ 0.05 [66]. Small-study effects include such things as publication bias and the overestimation of treatment effects in studies of lower quality. For primary outcomes, influence analysis was conducted in order to examine the effects of each study on the overall results. In addition, cumulative meta-analysis, ranked by year, was also conducted [67].

#### 2.6.3. Moderator Analysis

Mixed-effects, ANOVA-like models for meta-analysis were used to compare between-group differences (*Q*_*b*_) in FN and LS BMD according to selected categorical variables, assuming that each category included at least 2*g*'s. A random-effects model was used to combine studies within each subgroup while a fixed-effect model was used to combine subgroups and yield the overall *g*. Between-study variance (*τ* ^2^) was not assumed to be equal for all subgroups. *A priori* variables to examine included type of control group (nonintervention, other), matching (yes, no), risk of bias for sequence generation, allocation concealment, blinding, incomplete outcome data, selective outcome reporting (low versus high risk), type of analysis (intention to treat, per protocol), provision of sample size estimates (yes, no), whether the study was funded (yes, no), adverse events (yes, no), race/ethnicity, drugs, other than hormone therapy, which could positively or negatively affect BMD (yes, no), hormone therapy, including oral contraceptives (yes, no), rheumatoid arthritis (yes, no), cigarette smoking (yes, no), alcohol consumption (yes, no), changes in physical activity habits outside the exercise intervention (yes, no), whether calcium or vitamin D supplements were given during the study (yes, no), previous fractures (yes, no), type of exercise (aerobic, strength, both), exercise supervision status (supervised, unsupervised, both), location in which exercise took place (facility, home, both), exercise participation (self, group, both), reaction forces (ground, joint, both), and instrument used to assess BMD (Lunar, Hologic). However, because of a lack of data (<2*g*'s per category), moderator analysis was limited to type of control group, type of analysis, sample size estimates, funding (FN only), calcium administration during the study (FN only), type of exercise (aerobic, strength), exercise supervision (FN only), location in which exercise took place (facility versus home, FN only), exercise participation (group versus self, FN only), reaction forces (ground versus joint), and instrument used to assess BMD (FN only). *Post hoc*, an examination for potential differences in FN and LS BMD when partitioned according to whether studies were at a low versus unclear risk for incomplete outcome data was conducted. Because of a lack of data for categorizing, a statistical examination for other forms of bias (sequence generation, allocation concealment, blinding, selective outcome reporting) was not possible. The alpha level for statistical significance for *Q*_*b*_ was set at *P* ≤ 0.05.

#### 2.6.4. Metaregression

Simple mixed-effects, method of moments metaregression was used to examine the association between changes in FN and LS BMD and selected continuous variables, assuming that at least 3*g*'s were available for each analysis. Potential predictors established *a priori* included percentage of dropouts in the exercise intervention groups, age, length, frequency and intensity of training, duration of training (aerobic exercise only), compliance to the exercise protocol, total minutes of training (unadjusted and adjusted for compliance, aerobic exercise only), number of sets, repetitions and exercises (strength training only), load rating of the exercise interventions, calculated from previous research [51], baseline BMD and changes in cardiorespiratory fitness, balance (static and dynamic), calcium intake, muscular strength (upper and lower), body weight, BMI, lean body mass, fat mass, and percent body fat. However, because of a lack of data (<3*g*'s), metaregression analysis was limited to dropouts, age, length of training, frequency of training, duration of training, compliance, unadjusted total minutes of training, adjusted total minutes of training (FN only), load rating, number of sets and exercises (FN only), changes in upper and lower body strength, bodyweight (FN only), and baseline BMD. Analyses were limited to simple metaregression versus multiple metaregression because of missing data for different variables from different studies. The alpha level for statistical significance was set at *P* ≤ 0.05.

#### 2.6.5. Software Used for Statistical Analysis 

Data were analyzed using Comprehensive Meta-Analysis (version 2.2) [68], Microsoft Excel 2007 [56], and SSC-Stat (version 2.18) [69].

## 3. Results

### 3.1. Study Characteristics

After screening 1055 citations, seven studies representing 17 groups (10 exercise, 7 control) and 521 participants (269 exercise, 252 control) met the criteria for inclusion [14–20]. A flow diagram for the selection of studies is shown in Supplementary File 1, a general description of the characteristics of each study in Table 1, and baseline characteristics of the participants in Table 2. A list of excluded studies, including the reasons for exclusion, is available upon request from the corresponding author. For the included studies, the number of exercise groups exceeded the number of control groups because two studies included more than one exercise group [14, 17]. All studies were published in English-language journals between 1995 and 2011 [14–20]. Five studies were conducted in the United States [15, 17–20], one in Australia [14] and one in Finland [16]. For type of control groups, four studies used a nonintervention control group [16–18, 20] while three others used alternative approaches (usual care, attention control) [14, 15, 19]. With respect to matching, one study matched participants according to body weight and oral contraceptive use [16] while another matched according to age and oral contraceptive use [20]. None of the studies used a crossover design [14–20]. For sample size justification, three studies supplied power estimates to support such [14, 16, 19]. Five studies used the per-protocol approach [14, 15, 17, 18, 20] while the remaining two used intention to treat [16, 19] to analyze their data.

For external funding, five [15–17, 19, 20] of 7 studies reported receiving some type of external funding to conduct their project. The dropout rate ranged from 13.9% to 63.6% in the exercise groups ($\overset{-}{x}\pm \text{SD}=40.3\%\pm 17.8\%$, Mdn = 46%) and 5.0% to 57.8% in the control groups ($\overset{-}{x}\;\pm \text{SD}=28.5\%\pm 19.7\%$, Mdn = 28%). For the 4 studies that reported dropout data separately for exercise and control groups [14, 16, 17, 19] reasons for dropping out or being dropped in the exercise groups included changed circumstances, time constraints, injuries or pain which may or may not have been associated with the exercise intervention, personal issues, pregnancy, moving, loss of interest, uptake of medications that could affect BMD, and noncompliance with the exercise intervention. For control groups, reasons included changed circumstances, injury, moving, loss of interest, pregnancy, and uptake of medications that could affect BMD. For the one study that provided information, no serious adverse events were reported [16]. 

### 3.2. Participant Characteristics

 Initial physical characteristics of the participants are shown in Table 2. For the three studies that reported data on race/ethnicity [15, 18, 19], participants included primarily Whites. Other racial/ethnic groups included Asians as well as Hispanics and/or Latinos. Two studies reported that none of the subjects were taking any type of hormone therapy, including hormonal contraceptives [15, 18] while the other five reported that some were [14, 16, 17, 19, 20]. For drugs other than hormone therapy that could affect BMD, two studies reported no use of such [18, 20] while one reported that some were [16]. Three studies reported that none of the participants had osteopenia or osteoporosis [15, 17, 20] while two reported no secondary osteoporosis [15, 20]. With respect to cigarette smoking, two studies reported that none of the participants were currently smoking cigarettes [16, 17]. Three studies in which data were available reported no change in the participants' levels of exercise beyond the exercise intervention itself [16, 18, 19]. Two studies reported that calcium was given to all participants [17, 18]; one reported that some participants received calcium [15] while two others reported no calcium supplementation [14, 19]. For vitamin D intake, one study reported administering vitamin D to all participants [15] while two others reported no administration of vitamin D [14, 19]. 

### 3.3. Exercise Intervention Characteristics

A description of the training program characteristics is shown in Table 1. As can be seen, the exercise interventions varied. Across all intervention groups, length of training ranged from 24 to 104 weeks ($\overset{-}{x}\;\pm \text{SD}=63.6\pm 32.8$, Mdn = 65) while frequency ranged from 2 to 7 days per week ($\overset{-}{x}\pm \text{SD}=3.1\pm 1.4$, Mdn = 3). Compliance, defined as percentage of exercise sessions attended, ranged from 44% to 90% ($\overset{-}{x}\;\pm \text{SD}=71.7\%\pm 17.7\%$, Mdn = 83%). For those groups in which data were available, four participated in either supervised or unsupervised exercise while one participated in both. For location where exercise took place, six participated in facility-based exercise, three in home-based exercise, and one did both. With respect to exercise participation, three groups participated in group-based exercise, four participated in exercise on their own, and one did both. Five exercise groups participated in ground reaction force exercise, three in joint reaction force exercise, and two in both. The exercise load rating ranged from 9.1 to 1481 ($\overset{-}{x}\;\pm \text{SD}=388.2\pm 618.6$, Mdn = 10.1) for the nine groups that reported data for such.

### 3.4. BMD Assessment Characteristics

 A description of FN and LS BMD assessment is shown in Table 1. For those studies in which data were available, three reported using Lunar dual-energy X-ray absorptiometry [14, 19, 20] while two others used a Hologic instrument [15, 17]. Coefficients of variation ranged from 0.5% to 4% at the FN and 0.3% to 4% at the LS. 

### 3.5. Risk of Bias Assessment

**Figure 1:**
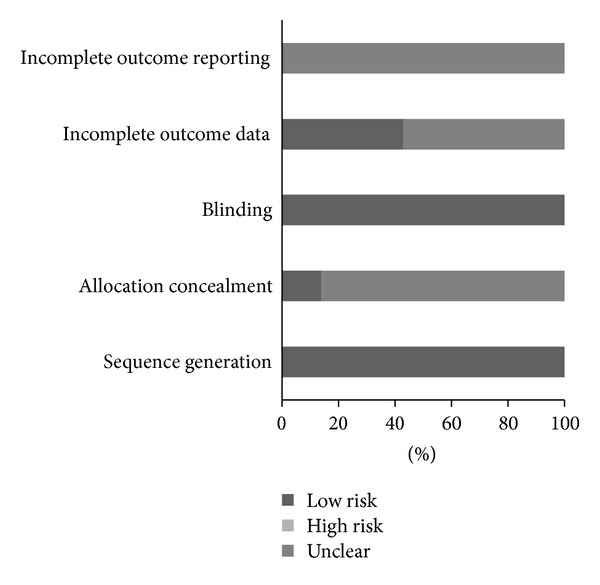
Risk of bias. Pooled risk of bias results using the Cochrane Risk of Bias Assessment Tool [57].

 Overall results for risk of bias are shown in Figure 1 while study level results are shown in Supplementary file 3. As can be seen, all studies were considered to be at a low risk for bias with respect to sequence generation and blinding [14–20]. In contrast, allocation concealment was categorized as unclear in 86% of the studies and low risk in 14%. Results for incomplete outcome data were mixed, with 43% considered to be at low risk for bias and 57% classified as unclear. Finally, because none of the studies provided a clinical trials registry number, selective outcome reporting was considered to be unclear for all of the studies [14–20]. 

### 3.6. Changes in Primary Outcomes

#### 3.6.1. Changes in FN BMD

**Figure 2:**
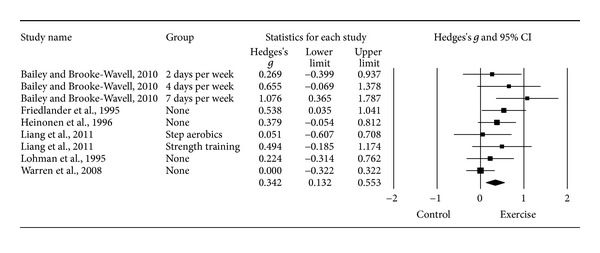
Forest plot for changes in FN BMD. Forest plot for point estimate standardized effect size changes (*g*) in FN BMD. The black squares represent the standardized mean difference (*g*) while the left and right extremes of the squares represent the corresponding 95% confidence intervals. The middle of the black diamond represents the overall standardized mean difference (*g*) while the left and right extremes of the diamond represent the corresponding 95% confidence intervals. Negative results favor control groups while positive results favor exercise groups.

**Figure 3:**
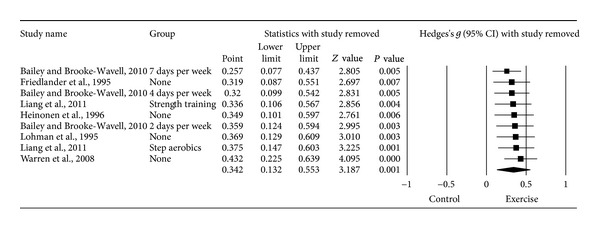
Influence analysis for changes in FN BMD. Influence analysis for point estimate standardized effect size changes (*g*) in FN BMD with each corresponding study deleted from the model once. The black squares represent the standardized mean difference (*g*) while the left and right extremes of the squares represent the corresponding 95% confidence intervals. The middle of the black diamond represents the overall standardized mean difference (*g*) while the left and right extremes of the diamond represent the corresponding 95% confidence intervals. Results are ordered from smallest to largest values of *g*. Negative results favor control groups while positive results favor exercise groups.

**Figure 4:**
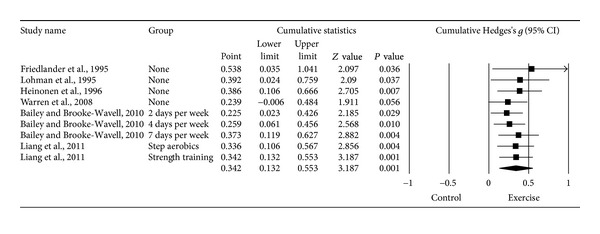
Cumulative meta-analysis for changes in FN BMD. Cumulative meta-analysis, ordered by year, for point estimate standardized effect size changes (*g*) in FN BMD. The black squares represent the standardized mean difference (*g*) while the left and right extremes of the squares represent the corresponding 95% confidence intervals. The results of each corresponding study are pooled with all studies preceding it. The middle of the black diamond represents the overall standardized mean difference (*g*) while the left and right extremes of the diamond represent the corresponding 95% confidence intervals. Negative results favor control groups while positive results favor exercise groups.

Ten *g*'s representing 521 participants from seven studies [14–20] resulted in a small but statistically significant benefit in FN BMD (*g* = 0.280, 95% CI = 0.036, 0.524, *P* = 0.03, *Q* = 17.8, *P* = 0.04, *I* ^2^ = 49.6%). However, one outlier was detected and deleted from all further FN BMD analyses [20]. With the one outlier deleted from the model, results remained small, statistically significant, and with a nonsignificant and small amount of heterogeneity observed (Table 3 and Figure 2). Changes were equivalent to a 1.1% benefit (0.4% increase in the exercise groups, −0.7% decrease in the control groups). The NNT was 5 while the 95% PI was −0.116 to 0.800. Statistically significant small-study effects were observed (*P* = 0.05). With each study deleted from the model once, results remained statistically significant (Figure 3). Cumulative meta-analysis demonstrated that results have been statistically significant, or there has been a trend for statistical significance, since inception of the publication of the first two studies in 1995 (Figure 4) [15, 18]. When results were collapsed so that only one *g* represented each study, increases in FN BMD remained small, statistically significant, and with a nonsignificant and small amount of heterogeneity (*g* = 0.323, 95% CI = 0.109, 0.537, *P* = 0.003, *Q* = 7.3, *P* = 0.20, *I* ^2^ = 31.4%). Because *g* was used, no missing data for FN BMD needed to be requested from the original study authors. The calculation of *g* was based on relative values from five studies [14–17, 20] and absolute values from the other two [18, 19]. Original metric change outcome SD's for exercise and control groups were estimated from change score SD's in three studies [15, 16, 20], one of which was transformed from sample sizes and standard errors of the means [20], 95% confidence intervals from two studies [14, 17], and initial and final standard deviations in two others [18, 19]. 

#### 3.6.2. Moderator Analysis for FN BMD

The moderator analyses for FN BMD are shown in Supplementary File 4. As can be seen, there was a trend for greater benefits in FN BMD for those studies published in countries other than the United States. In addition, there was a trend for greater benefits in those participating in home versus facility-based exercise. No other statistically significant differences for FN BMD were observed, including when reporting of incomplete outcome data were partitioned according to low versus unclear risk (*Q*_*b*_ = 0.55, *P* = 0.46).

#### 3.6.3. Regression Analysis for FN BMD

Simple metaregression results for changes in FN BMD are shown in Supplementary File 5. As can be seen, there was a statistically significant and positive relationship between benefits in FN BMD and the number of sets performed when resistance training while an inverse relationship was observed for exercise frequency. A trend for statistical significance was observed for greater benefits in FN BMD and (1) shorter exercise interventions, (2) lower initial FN BMD, (3) increases in body weight, and (4) decreases in upper body strength. 

#### 3.6.4. Changes in LS BMD

**Figure 5:**
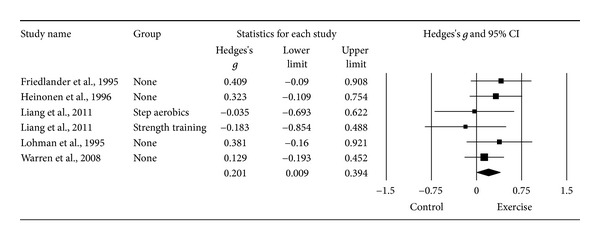
Forest plot for changes in LS BMD. Forest plot for point estimate standardized effect size changes (*g*) in LS BMD. The black squares represent the standardized mean difference (*g*) while the left and right extremes of the squares represent the corresponding 95% confidence intervals. The middle of the black diamond represents the overall standardized mean difference (*g*) while the left and right extremes of the diamond represent the corresponding 95% confidence intervals. Negative results favor control groups while positive results favor exercise groups.

**Figure 6:**
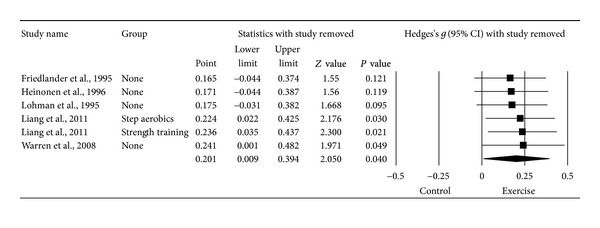
Influence analysis for changes in LS BMD. Influence analysis for point estimate standardized effect size changes (*g*) in LS BMD with each corresponding study deleted from the model once. The black squares represent the standardized mean difference (*g*) while the left and right extremes of the squares represent the corresponding 95% confidence intervals. The middle of the black diamond represents the overall standardized mean difference (*g*) while the left and right extremes of the diamond represent the corresponding 95% confidence intervals. Results are ordered from smallest to largest values of *g*. Negative results favor control groups while positive results favor exercise groups.

**Figure 7:**
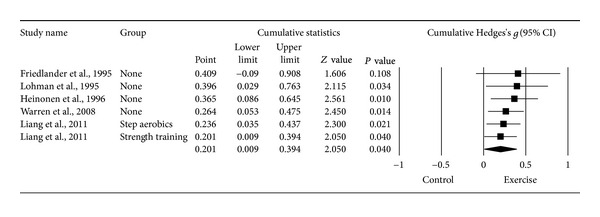
Cumulative meta-analysis for changes in LS BMD. Cumulative meta-analysis, ordered by year, for point estimate standardized effect size changes (*g*) in LS BMD. The black squares represent the standardized mean difference (*g*) while the left and right extremes of the squares represent the corresponding 95% confidence intervals. The results of each corresponding study are pooled with all studies preceding it. The middle of the black diamond represents the overall standardized mean difference (*g*) while the left and right extremes of the diamond represent the corresponding 95% confidence intervals. Negative results favor control groups while positive results favor exercise groups.

Seven gs representing 457 participants from six studies [15–20] resulted in a trivial and non-significant difference in LS BMD (*g* = 0.115, 95% CI = −0.108, 0.339, *P* = 0.31, *Q* = 8.5, *P* = 0.20, *I* ^2^ = 29.5%). However, the same outlier as for FN BMD was detected and deleted from all further LS BMD analyses [20]. With the one outlier deleted, results were small but statistically significant and heterogeneity (*I* ^2^) was reduced to 0% (Table 3 and Figure 5). The NNT was 9 while the 95% PI was −0.071 to 0.473. Calculation of percent change was not possible because of missing data from two studies [16, 19]. No statistically significant small-study effects were observed (*P* = 0.034). With each study deleted from the model once, results were no longer statistically significant or there was no longer a trend for statistical significance when two were deleted from the model (Figure 6) [15, 16]. Cumulative meta-analysis demonstrated that results have been statistically significant since inception of the second study in 1995 (Figure 7) [18]. When results were collapsed so that only one *g* represented each study, increases in LS BMD remained small, statistically significant, and with no apparent statistical heterogeneity (*g* = 0.201, 95% CI = 0.009, 0.394, *P* = 0.04, *Q* = 3.2, *P* = 0.52, *I* ^2^ = 0%). Because *g* was used, no missing data for LS BMD needed to be requested from the original study authors. The calculation of *g* was based on relative values from four studies [15–17, 20] and absolute values from the other two [18, 19]. Original metric change outcome SD's for exercise and control groups were estimated from change score SD's in three studies [15, 16, 20], one of which was transformed from standard errors of the means [20], 95% confidence intervals from two studies [17], and initial and final standard deviations in two others [18, 19]. 

#### 3.6.5. Moderator Analysis for LS BMD

Moderator analyses for LS BMD are shown in Supplementary File 4. As can be seen, no statistically significant differences were observed, including when the reporting of incomplete outcome data were partitioned according to low versus unclear risk (*Q*_*b*_ = 0.43, *P* = 0.51). 

#### 3.6.6. Regression Analysis for LS BMD

Simple metaregression results for changes in LS BMD are shown in Supplementary File 5. As shown, no statistically significant associations were observed. A trend for a statistically significant association was observed for greater benefits in LS BMD and earlier published studies. 

### 3.7. Changes in Secondary Outcomes

The overall results for secondary outcomes are shown in Table 3. No statistically significant differences were found for BMD at Ward's triangle and the trochanteric regions as well as for bodyweight. Small but statistically significant increases were observed for both upper and lower body strength. A trend for a statistically significant and moderate amount of heterogeneity was observed for changes in lower body strength. For both upper and lower body strength, the NNT was 4 while the 95% PI was −0.879 to 1.850 for upper body strength and −0.492 to 1.388 for lower body strength. Small-study effects were non-significant for changes in strength in both the upper (*P* = 0.33) and lower (*P* = 0.70) body. When results were collapsed so that only one *g* represented each study, increases in lower body strength remained small, statistically significant, and with no apparent heterogeneity (*g* = 0.429, 95% CI = 0.237, 0.622, *P* = 4.37, *Q* = 1.4, *P* = 0.71, *I* ^2^ = 0%). No study level analysis was needed for changes in upper body strength because none of the studies included multiple groups.

## 4. Discussion

 The primary purpose of meta-analysis is to reach general conclusions regarding a body of research [70]. The primary purpose of this study was to use the aggregate data meta-analytic approach to determine the effects of exercise on FN and LS BMD in premenopausal women and to examine potential moderators and predictors of such changes. To the best of the investigative team's knowledge, this is the first meta-analysis on exercise and BMD in premenopausal women limited to randomized controlled trials. The overall findings suggest that exercise results in small, as defined by Cohen's categorization for the magnitude of effect for *g* [60], but statistically significant benefits in both FN and LS BMD. These findings are similar to the statistically significant results reported for LS BMD in two earlier meta-analyses but differ with respect to FN BMD [37, 44]. One possible reason for the lack of statistically significant findings for FN BMD in the two previous meta-analyses may have to do with the small number of results that were pooled. Specifically, one meta-analysis pooled results from three randomized controlled trials [44] while a second pooled results from five randomized controlled trials [37]. A second possible reason may have to do with the differing inclusion criteria across meta-analyses. In contrast, the overall findings of the current investigation are in agreement with the overall findings of the James and Carroll meta-analysis [41].

 To the best of the investigative team's knowledge, this is the first meta-analysis to report NNT for exercise and BMD studies in premenopausal women. The current findings suggest that less than 10 women would need to exercise in order to derive benefit in BMD at the FN and LS. However, whether the magnitude of effect is large enough to reduce the risk of site-specific fractures in those women who improve their FN and LS BMD is not known. 

 While the exercise-induced benefits observed for FN and LS BMD were considered small and statistically significant, the direct clinical importance of such changes is not known. Previous meta-analytic work in postmenopausal women reported that a 1% improvement in spine BMD was associated with a small but statistically significant 0.03 decrease in the relative risk of vertebral fracture as a result of antiresorptive therapy [71]. However, this study was limited to postmenopausal women using antiresorptive agents. Since the effects of exercise on BMD may be different from antiresorptive therapy, these findings may need to be interpreted with caution when applied to exercise. While additional research is needed, it would seem plausible that any exercise-induced benefit on FN and LS BMD in premenopausal women might be beneficial, especially when viewed from a population-wide perspective. 

 While the overall results suggest that exercise benefits FN and LS BMD in premenopausal women, these findings should be viewed with respect to several factors. First, the 95% PI for treatment effects if a new trial was conducted crossed zero (0) for both FN and LS BMD. It has been suggested that nonoverlapping PI allows for more robust meta-analytic conclusions [64]. Second, small-study effects were observed for ES changes in FN BMD. This suggests that ES benefits may be inflated. Third, influence analysis for ES changes in LS BMD resulted in *P* values >0.10 when two studies were deleted separately from the model. This suggests a possible lack of robustness across studies. Finally, while BMD has been shown to account for approximately 60% to 70% of the variation in bone strength, it does not account for other aspects of bone quality such as microarchitecture [72, 73]. Thus, the potential benefits of effects of exercise on bone strength, when limited to BMD, may be underestimated. However, a recent systematic review with meta-analysis was only able to locate one randomized controlled trial addressing the effects of exercise on bone outcomes other than BMD (bone strength index, stress-strain index, maximal moment of inertia, cross-sectional moment of inertia, and section moduli) in premenopausal women [74]. Overall, no statistically significant effect of a 12-month progressive impact exercise program was found at the proximal tibia and femoral shaft [75]. However, greater compliance was associated with improvements ranging from 0.5% to 2.5% at the proximal tibia [75]. Clearly, additional well-designed randomized controlled trials are needed to address the effects of exercise on bone outcomes other than BMD. 

 Moderator analyses resulted in a trend for greater benefits on FN BMD when exercise took place in the home versus a facility. Since the investigative team is not aware of any consensus in the literature regarding which location is superior, future research in this area appears warranted. In addition to several other non-significant findings, no statistically significant differences were observed when data were partitioned according to type of exercise as well as type of reaction forces induced by exercise. 

 In subgroup analyses, a recent meta-analysis by James and Carroll reported changes in FN and LS BMD for high-impact only protocols as well as combined impact/resistance training protocols in premenopausal women [41]. A significant improvement in FN but not LS was found as a result of high-impact protocols while combined impact/resistance training resulted in significant improvements in LS but not FN BMD [41]. When limited to ground reaction force exercise, the results of the current meta-analysis are similar to the high-impact protocol results of James and Carroll [41] (FN, *g* = 0.454, 95% CI = 0.143, 0.764, *P* = 0.004; LS, *g* = 0.215, 95% CI = −0.146, 0.576, *P* = 0.243). However, because of the small sample size, investigators in the current meta-analysis were unable to perform subgroup analyses for combined ground and joint reaction force exercise. While these findings are interesting, it is probably not appropriate to make a decision about whether ground and joint reaction force exercise studies should be pooled based on running separate analyses for each. The primary reasons for this include the small sample sizes as well as the inability to control for other potentially confounding variables. Rather, these potential differences would need to be tested in well-designed randomized controlled trials. 

 Simple metaregression analyses resulted in several noteworthy associations that may be appropriate for future investigation. Specifically, there was a trend for greater increases in FN BMD with shorter exercise interventions as well as a statistically significant association between increases in FN BMD and fewer days per week of exercise. One possible explanation for the negative associations observed may have to do with the loss of calcium from excessive exercise [76, 77]. This causes a decrease in serum calcium, followed by an increase in serum parathyroid hormone, which then stimulates bone resorption [76, 77]. However, no association was observed between changes in FN BMD and duration of training as well as exercise load rating. Thus, while these findings are interesting, further dose-response research is needed before any firm conclusions can be drawn. For resistance training, greater increases in FN BMD were associated with a greater number of sets. Since sweating as a result of resistance training is usually not as great as that from aerobic exercise, it may be that a greater but undetermined amount of resistance training is needed to increase FN BMD in premenopausal women. However, no association was found between the number of exercises performed and changes in FN BMD. Given the former, it would appear appropriate to suggest that future dose-response studies are needed to address this issue. Until that time, it would appear plausible to suggest adherence to current exercise guidelines for optimizing BMD in adults [78]. 

 The trend for greater benefits in FN BMD and lower baseline BMD at the FN suggests that those with lower FN BMD may derive the greatest benefits as a result of exercise. This finding would seem to be entirely reasonable. The trend for increases in FN BMD to be associated with increases in body weight supports well-established research regarding greater BMD in heavier adult humans. Other than chance, the investigative team has no plausible explanation for the observed association between increases in FN BMD and smaller increases in upper body strength. Finally, there was a trend for greater benefits in LS BMD for those studies published during the earlier years. This observed association may be reflective of improved study designs in more recent years. 

 While the results for moderator and regression analyses are interesting, they should be viewed with respect to the following potential limitations. First, because of missing data for different variables from different studies, multiple metaregression analysis was not performed. Thus, controlling for potential confounding factors was not possible. Second, because of the large number of statistical tests conducted, one or more of the significant findings may have been nothing more than the play of chance. However, no adjustment was made for alpha values because such adjustments tend to be overly conservative [79]. In addition, the investigative team did not want to miss any potentially important findings that might be worthy of further investigation [79]. Third, since potential moderators and predictors are not randomly assigned in meta-analysis, such analyses are considered to be observational [80]. Therefore, causal inferences cannot be derived [80]. However, such differences and associations do provide direction for future research.

 For secondary outcomes, statistically significant increases in both upper and lower body strength were observed. This suggests that exercise, particularly resistance training exercise, can improve both upper and lower body strength in premenopausal women. This observation demonstrates two of the many benefits that can be derived from a regular exercise program [81]. However, results for secondary outcomes in any meta-analysis need to be interpreted with caution since the inclusion of such are not mandatory for inclusion in a meta-analysis. Thus, secondary outcomes may represent a potentially biased sample of results.

 Several suggestions in relation to the conduct and reporting of future randomized controlled trials on the effects of exercise in premenopausal women appear appropriate.

 The first issue has to do with the risk of bias findings. For example, while all of the studies were considered to be at a low risk of bias with respect to randomized sequence generation, all but one study [15] was considered to be at an unclear risk for adequate allocation concealment. While randomized sequence generation is important, it might be ineffective if it is not protected by adequate concealment of the allocation from those responsible for enrolling and assigning participants [82]. To support this contention, Pildal et al. [83] reported that binary effect estimates from randomized controlled trials with inadequate allocation concealment were approximately 18% more beneficial than estimates from trials with adequate concealment. However, a more specific analysis by Wood et al. [84] found that intervention effect estimates were inflated when inadequate allocation concealment was present in trials with a subjective outcome but not when the outcome was objective. Given that the primary outcomes in the current meta-analysis were objective measures, that is, changes in FN and LS BMD, inadequate sequence generation may not have posed much of a threat. Notwithstanding the former, it would still seem plausible to suggest that future studies perform appropriate allocation concealment procedures and report this information in their published work.

 Because of the objective nature of BMD assessment, all studies were considered to be at a low risk of bias for blinding. While this may indeed be the case, it is also possible that such a classification may not have been appropriate. For example, Pildal et al. [83] reported that a lack of blinding in randomized controlled trials was associated with exaggerated odds ratios averaging 9%. However, this potential form of bias has been reported to be greater for trials with more subjective versus objective outcomes [84]. Thus, blinding as a potential form of bias may not have posed much of a threat in the current meta-analysis. This is important since it is extremely difficult to adequately blind participants enrolled in exercise intervention studies. Regardless, it would seem appropriate to recommend that investigators do the best that they can to blind all relevant parties to group assignment. 

 Incomplete (missing) outcome data due to drop outs during a study and/or exclusions from a study may result in biased effect estimates [82]. For the current meta-analysis, three studies were considered to be at a low risk for bias [15, 16, 19] while four were classified as unclear risk [14, 17, 18, 20]. However, since no statistically significant differences between the two were found for changes in FN and LS BMD, this potential form of bias did not seem to have an effect in the current meta-analysis. 

 Selective outcome reporting may be considered as a subset of findings that are reported based on their results [85]. The major concern is that results which are not statistically significant may be withheld. As a result, meta-analyses may overestimate treatment effects. To support this potential form of bias, at least three studies have shown that outcomes with statistically significant findings are more likely to be reported than outcomes with non-significant results [86–88]. For the current meta-analysis, all of the studies were classified as being at an unclear risk of bias for selective outcome reporting. This was based on the fact that none of the studies provided a clinical trials registry number so that the investigative team could retrieve and review the original study protocol. Given the inability to determine such, this potential form of bias cannot be ruled out for the current meta-analysis. It is strongly suggested that future studies report their clinical trials registry number so this potential form of bias can be determined. However, recent research by Hartling et al. [89], has suggested that the search and identification for study protocols to assess selective outcome reporting bias may not be feasible or productive. Given the former, they suggest that in the absence of study protocols that the outcomes reported in the methods section of a paper should be compared with those reported in the results [89]. 

 Future randomized controlled trials should also report more detailed information, by group, for race/ethnicity, dropouts, adverse events, cigarette smoking, alcohol consumption, pharmacological intake, parental history of osteoporosis and fractures, changes in physical activity habits outside the exercise intervention as well as baseline and final changes in cardiorespiratory fitness, static and dynamic balance, calcium and vitamin D levels, fat mass, and lean body mass. In addition, it is suggested that future studies analyze and report data using both per-protocol and intention-to-treat analyses. This would allow one to determine both the efficacy (per-protocol analysis) and effectiveness (intention-to-treat analysis) of exercise on FN and LS BMD in premenopausal women. 

## 5. Conclusions

 The primary and accomplished aim of this study was to use the meta-analytic approach to determine the overall effects of ground and joint reaction exercise on FN and LS BMD in premenopausal women when limited to randomized controlled trials. The overall findings of the current meta-analysis provide additional support regarding the benefits of exercise, including NNT estimates to aid decision makers regarding the utility of exercise for improving FN and LS BMD in premenopausal women. In addition, this study provides first-time meta-analytic evidence, when limited to randomized controlled trials, of potential moderators and predictors with respect to changes in FN and LS BMD, which appears worthy of pursuing in future well-designed randomized controlled trials. The inability of the current meta-analysis to provide a definitive exercise prescription warrants further research. In addition, the results should be interpreted with some trepidation given that the quality of evidence could be improved.

## Supplementary Material

Supplementary File 1: Flowchart describing the results of the search process, screening and study eligibility decisions.
Supplementary File 2: Search strategy for CINAHL database.
Supplementary File 3: Risk of bias (study level).
Supplementary File 4: Moderator analyses for FN and LS BMD.
Supplementary File 5: Table of Meta-regression results for changes in FN and LS BMD.

## Figures and Tables

**Table 1:** General characteristics of studies.

Study	Country	Participants	Exercise intervention	BMD Assessment
Bailey and Brooke-Wavell, 2010 [14]	United Kingdom	85 healthy, premenopausal women 18 to 45 yrs of age assigned to 0 (*n* = 20), 2 (*n* = 21), 4 (*n* = 22), or 7 (*n* = 22) days/wk of exercise	2, 4, or 7 days/wk of 5 sets of 10 hops on one limb with 15 seconds of walking between each set for 6 months	DEXA (GE Lunar Prodigy Advance) at the FN

Friedlander et al., 1995 [15]	United States	63 women 20 to 35 yrs of age assigned to either an exercise (*n* = 32) or stretching (*n* = 31) group	3 days/wk, 1 h/session, alternating classes of circuit training, strength training, and aerobic exercise (70–85% of VO_2 max⁡_), for 2 yrs	DEXA (Hologic QDR 1000) at the LS & FN

Heinonen et al., 1996 [16]	Finland	84 healthy, sedentary premenopausal women 35 to 40 yrs of age assigned to either a training (*n* = 39) or control (*n* = 45) group	3 days/wk, 1 h/session (15 min warm-up, 20 min high- impact jump training, 15 min calisthenics, 10 min cool down), for 18 months	DEXA (Norland XR-26) at the LS & FN

Liang et al., 2011 [17]	United States	51 healthy, untrained women 20 to 35 yrs of age assigned to a strength training (*n* = 15), step aerobics (*n* = 16), or control (*n* = 20) group	3 days/wk, 40 min/session, strength: 1–3 sets, 8–15 reps, 65–80% 1RM, 8 exercises; step aerobics: step, hop, walk, run in place, 20 cm step height, 15–300 hop cycles/session, for 12 months	DEXA (Hologic QDR 4500W)

Lohman et al., 1995 [18]	United States	56 premenopausal women 28 to 39 yrs of age assigned to either an exercise (*n* = 22) or control (*n* = 34) group	3 days/wk, 1 h/session, 3 sets, 8–12 reps, 70–80% 1RM, 12 weight lifting exercises, 18 months	DEXA (Lunar DPX) at the LS & FN

Warren et al., 2008 [19]	United States	148 healthy, sedentary, overweight premenopausal women 25 to 44 yrs of age assigned to either an exercise (*n* = 72) or control (*n* = 76) group	2 days/wk, strength training, 3 sets, 8–10 reps, for 2 yrs	DEXA (Lunar Prodigy) at the LS & FN

Weaver et al., 2001 [20]	United States	55 women 18 to 31 yrs of age assigned to either an exercise (*n* = 28) or control (*n* = 27) group	3 days/wk of super circuit resistance training, 8 upper and 8 lower body exercises with a cycle ergometer between each station, 8–12 reps, 70% 1RM, plus 60 min of jumping rope/wk, for 24 months	DEXA (DXA Lunar) at the LS & FN

BMD: bone mineral density; DEXA: dual-energy X-ray absorptiometry; FN: femoral neck; LS: lumbar spine; yrs: years; min: minute(s); h: hour(s); wks: weeks; wk: week; RM: repetition maximum; reps: repetitions; VO_2 max⁡_: maximum oxygen consumption; description of groups is limited to those that met the inclusion criteria for the current meta-analysis; description of BMD assessment is limited to the primary outcomes of the current meta-analysis (FN and LS). Number of participants is limited to those in which final BMD assessments were available.

**Table 2:** Initial physical characteristics of participants.

Variable	Exercise					Control				
	Groups (#)	Participants (#)	$\overset{-}{x}\pm \text{SD}$	Mdn	Range	Groups (#)	Participants (#)	$\overset{-}{x}\pm \text{SD}$	Mdn	Range
Age (yrs)	10	269	30.7 ± 5.5	31	23–39	7	252	32.8 ± 5.2	34	24–39
Body weight (kg)	10	269	62.1 ± 8.1	60	55–82	7	252	65.3 ± 7.5	63	58–81
BMD (g/cm^2^)										
Femoral neck	7	224	0.927 ± 0.085	0.840	0.85–1.070	6	233	0.938 ± 0.105	0.909	0.840–1.090
Lumbar spine	7	224	1.118 ± 0.120	1.080	0.991–1.290	6	233	1.145 ± 0.138	1.145	0.986–1.30
Ward's triangle	4	81	0.882 ± 0.062	0.863	0.883–0.970	3	81	0.911 ± 0.082	0.896	0.833–0.970
Trochanteric	6	196	0.775 ± 0.099	0.735	0.688–0.939	5	206	0.786 ± 0.10	0.765	0.690–0.909

Groups (#): number of groups in which data were available; participants (#): number of participants nested within groups; $\overset{-}{x}$± SD: mean ± standard deviation; Mdn: median; BMD: bone mineral density.

**Table 3:** Changes in primary and secondary outcomes.

Variable^a^	Studies (#)	ES (#)	Participants (#)	$\overset{-}{x}$ (95% CI)	*Z* (*P*)	*Q* (*P*)	*I* ^2^ (%)
Primary							
Femoral neck	7	9	466	0.342 (0.132, 0.553)	**3.19** (0.001)*	10.8 (0.22)	25.7
Lumbar spine	5	6	402	0.201 (0.009, 0.394)	**2.05** (0.04)*	3.3 (0.65)	0
Secondary							
Ward's triangle	3	4	162	0.088 (−0.207, 0.383)	0.59 (0.56)	2.9(0.41)	0
Trochanteric	7	10	521	0.085 (−0.097, 0.267)	0.92 (0.36)	10.5 (0.31)	14.1
Body weight (kg)	5	5	296	0.4 (−0.5, 1.3)	0.93 (0.35)	2.1 (0.72)	0
Strength (upper body)	3	3	295	0.49 (0.28, 0.70)	**4.56** (0.0001)*	1.2 (0.56)	0
Strength (lower body)	4	5	346	0.45 (0.14, 0.75)	**2.88** (0.004)*	**8.78** (0.07)**	54.4

^a^Unless noted otherwise, all outcomes are reported as standardized effect size (*g*); ES: effect size; #: number; participants (#): number of exercise and control participants nested within ES's and studies; *Z* (*P*): *Z*  score and alpha value; *Q* (*P*): Cochran's *Q* statistic and alpha value; *I* ^2^ (%): *I* squared; *statistically significant (*P* ≤ 0.05); **trend for statistical significance (*P* > 0.05 to ≤0.10).
