# Erratum to “Exercise and Bone Mineral Density in Premenopausal Women: A Meta-Analysis of Randomized Controlled Trials”

**DOI:** 10.1155/2013/583217

**Published:** 2013-09-19

**Authors:** George A. Kelley, Kristi S. Kelley, Wendy M. Kohrt

**Affiliations:** ^1^Meta-Analytic Research Group, Department of Biostatistics, School of Public Health, Robert C. Byrd Health Sciences Center, West Virginia University, P.O. Box 9190, Morgantown, WV 26506-9190, USA; ^2^Division of Geriatric Medicine, University of Colorado Denver, Anschutz Medical Campus, P.O. Box 6511, Mail Stop B179, 12631 East 17th Avenue-L15, Aurora, CO 80045, USA

The country name mentioned before reference [14] on page 5, column 2, line 3, should be the United Kingdom, not Australia. Also, the *P* value should be 0.68, not 0.034, on page 8, column 1, paragraph 2, line 12. None of these corrections affect any of the conclusions drawn.

