# Supplementary material for: Exercise and Bone Mineral Density in Premenopausal Women: A Meta-Analysis of Randomized Controlled Trials
Source: Int J Endocrinol. 2013 Jan 17;2013:741639. doi: 10.1155/2013/741639 (PMC3563173; doi:10.1155/2013/741639)
Supplement: Supplementary file 1 — Flowchart describing the results of the search process, screening and study eligibility decisions. Supplementary File 2: Search strategy for CINAHL database. Supplementary File 3: Risk of bias (study level). Supplementary File 4: Moderator analyses for FN and LS BMD. Supplementary File 5: Table of Meta-regression results for changes in FN and LS BMD. [file 741639.f1.pdf]

Supplementary File 1. Flow diagram for the selection of studies.

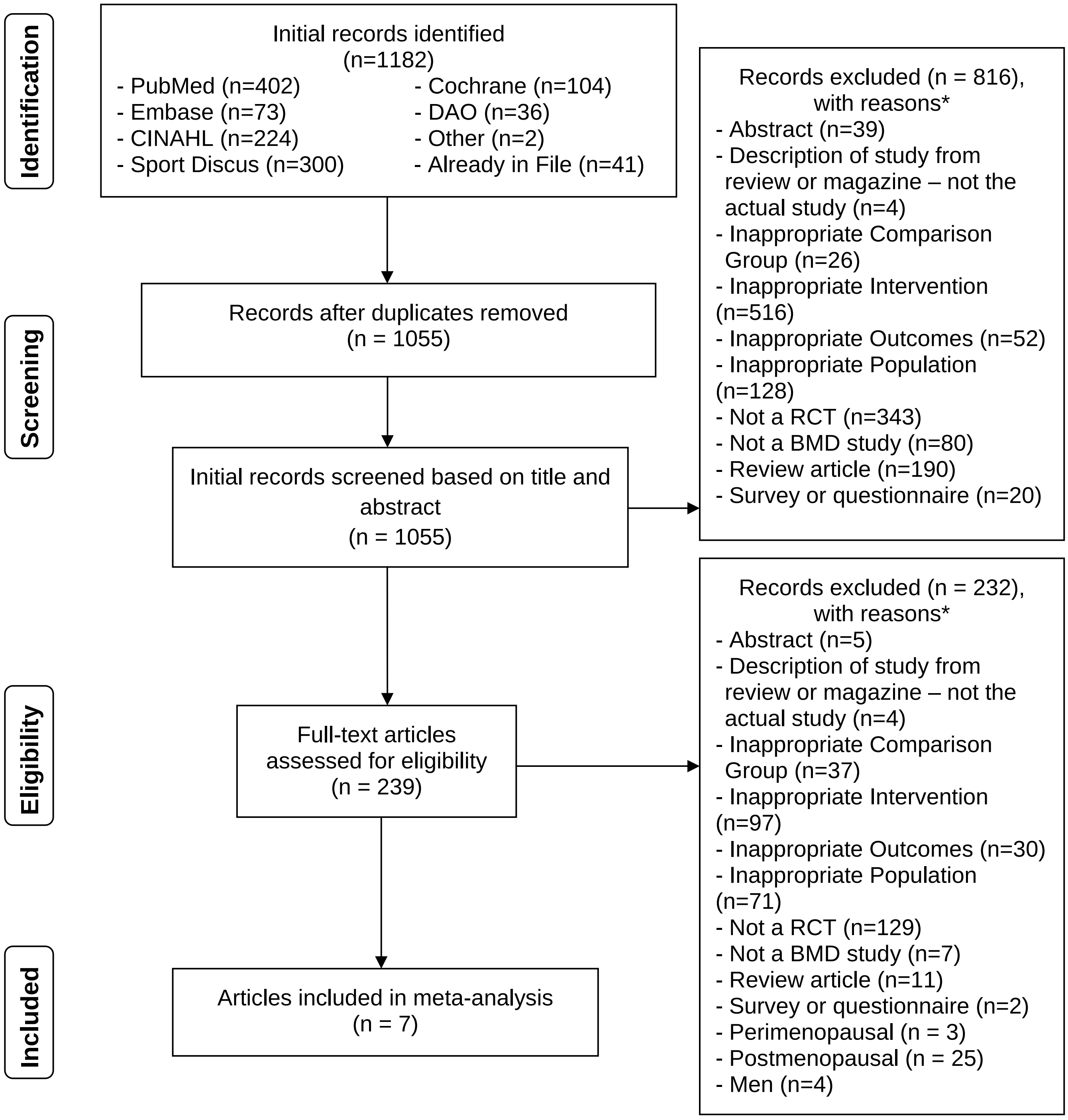

Supplementary File 2. Search strategy for CINAHL database.

Query Limiters/Expanders Last Run Via Results

S7 (s3 and s6) Search modes - Find all my search terms Interface -

EBSCOhost

Search Screen - Advanced Search

Database - CINAHL with Full Text 224

S6 (s4 or s5) Search modes - Find all my search terms Interface -

EBSCOhost

Search Screen - Advanced Search

Database - CINAHL with Full Text 41070

S5 (MH "Clinical Trials+") Limiters - Published Date from:

19890101-20100631; Human; Age Groups: All Adult

Search modes - Find all my search terms Interface - EBSCOhost

Search Screen - Advanced Search

Database - CINAHL with Full Text 35755

S4 TX random* w1 control* Limiters - Published Date from:

19890101-20100631; Human; Age Groups: All Adult

Search modes - Find all my search terms Interface - EBSCOhost

Search Screen - Advanced Search

Database - CINAHL with Full Text 13913

S3 (s1 and s2) Limiters - Published Date from: 19890101-20100631; Human;

Age Groups: All Adult

Search modes - Find all my search terms Interface - EBSCOhost

Search Screen - Advanced Search

Database - CINAHL with Full Text 672

S2 (NH "bone density") or TX bone w1 densit* Limiters - Published Date from: 19890101-20100631; Human; Age Groups: All Adult

Search modes - Find all my search terms Interface - EBSCOhost

Search Screen - Advanced Search

Database - CINAHL with Full Text 2392

S1 MH exercise or TX exercise Limiters - Published Date from: 19890101-20100631; Human; Age Groups: All Adult

Search modes - Find all my search terms Interface - EBSCOhost

Search Screen - Advanced Search

Database - CINAHL with Full Text 29586

Supplementary File 3. Risk of bias (study level).

Study	Sequence generation	Allocation concealment	Blinding	Incomplete outcome data	Selective outcome reporting
Bailey & Brooke-Wavell [14]	low	unclear	low	unclear	unclear
Friedlander et al. [15]	low	low	low	low	unclear
Heinonen et al. [16]	low	unclear	low	low	unclear
Liang et al. [17]	low	unclear	low	unclear	unclear
Lohman et al. [18]	low	unclear	low	unclear	unclear
Warren et al. [19]	low	unclear	low	low	unclear
Weaver et al. [20]	low	unclear	low	unclear	unclear

Notes: low, low risk of bias; unclear, unclear risk of bias; all studies considered to be at low risk of bias for blinding because of the objective nature of bone mineral density assessment.

Supplementary File 4. Moderator analyses for FN and LS BMD.

	FN				LS			
Variable	ES (#)	Participants (#)	$\bar{X}$ ± SE	$Q_b(p)$	ES (#)	Participants (#)	$\bar{X}$ ± SE	$Q_b(p)$
Country								
- USA	5	318	0.194 ± 0.113	**2.92 (0.09)****	--	--	--	--
- Other	4	148	0.535 ± 0.164		--	--	--	
Type of Control								
- Nonintervention	4	191	0.300 ± 0.140		4	191	0.191 ± 0.140	0.01 (0.92)
- Other	5	275	0.443 ± 0.197	0.35 (0.55)	2	211	0.212 ± 0.138	
Type of analysis								
- Per protocol	7	234	0.448 ± 0.120	1.66 (0.20)	4	170	0.205 ± 0.147	0.001 (0.97)
- Intention to treat	2	232	0.161 ± 0.187		2	232	0.199 ± 0.132	
Sample size estimate								
- Yes	5	296	0.398 ± 0.182	1.05 (0.82)	4	232	0.199 ±0.132	1.001 (0.97)
- No	4	170	0.344 ± 0.148		2	170	0.205 ± 0.147	
Funding for study								
- Yes	5	346	0.244 ± 0.118	1.40 (0.24)	--	--	--	--
- No	4	120	0.515 ± 0.196		--	--	--	
Calcium administered								
- Yes	3	107	0.247 ± 0.181	0.37 (0.54)	--	--	--	--

- No	4	212	0.437 + 0.254		--	--	--	
Type of exercise								
- Aerobic	2	120	0.280 + 0.184	0.15 (0.70)	3	120	0.215 + 0.184	0.11 (0.74)
- Strength	6	283	0.374 + 0.163		2	219	0.140 + 0.131	
Exercise supervision								
- Supervised	5	254	0.355 + 0.123	1.26 (0.26)	--	--	--	--
- Unsupervised	3	64	0.654 + 0.236		--	--	--	
Exercise location								
- Facility	6	402	0.228 + 0.098	**2.77 (0.10)****	--	--	--	--
- Home	3	64	0.654 + 0.236		--	--	--	
Exercise participation								
- Group	3	183	0.368 + 0.150	1.05 (0.31)	--	--	--	--
- Self	3	64	0.654 + 0.236		--	--	--	
Reaction forces								
- Ground	5	184	0.454 + 0.159	2.62 (0.11)	2	120	0.215 + 0.184	0.11 (0.74)
- Joint	3	219	0.121 + 0.131		3	219	0.140 + 0.131	
Instrumentation								
- Hologic	3	114	0.393 + 0.176	0.02 (0.89)	--	--	--	--
- Lunar	4	212	0.437 + 0.254		--	--	--	

Notes: Data reported as standardized effect size (*g*); FN, femoral neck; LS, lumbar spine; BMD, bone mineral density; ES(#),number of effect sizes; Participants(#), number of exercise and control participants nested within ES's; Z(*p*), *z*-score and alpha value; $\bar{X} \pm$ SE, mean $\pm$ standard error; $Q_b(p)$, between-group difference (Q_b) and alpha value (*p*); --, Insufficient data reported to calculate (< 2 ES's in one or more categories); **, trend for statistical significance ($p > 0.05$ to ≤ 0.10).

Supplementary File 5. Table of Meta-regression results for changes in FN and LS BMD.

	FN				LS			
Variable	ES (#)	Participants (#)	$\beta_1 \pm$ SE	*Z(p)*	ES (#)	Participants (#)	$\beta_1 \pm$ SE	Z(*p*)
Year of publication	9	466	-0.001 ± 0.016	-0.07(0.94)	6	402	-0.024 ± 0.014	**-1.70(0.09)****
Dropouts (%)	9	466	0.003 ± 0.006	0.53(0.59)	6	402	-0.001 ± 0.005	0.27(0.79)
Age (years)	9	466	-0.007 ± 0.023	-0.31(0.76)	6	402	0.014 ± 0.018	0.78(0.44)
Exercise length (wks)	9	466	-0.005 ± 0.003	**-1.74(0.08)****	6	402	0.004 ± 0.005	0.78(0.44)
Exercise frequency (days/wk)	9	466	0.200 ± 0.074	**-2.67(0.008)***	6	402	0.196 ± 0.230	0.85(0.39)
Exercise duration (min/session)[a]	3	183	-0.002 ± 0.018	-0.10(0.92)	3	183	-0.004 ± 0.016	-0.25(0.80)
Exercise compliance (%)	7	415	0.011 ± 0.013	0.93(0.35)	4	351	0.005 ± 0.012	0.39(0.70)
Total exercise (min)[a]	3	183	-0.004 ± 0.006	-0.68(0.50)	3	183	-0.004 ± 0.006	-0.78(0.43)
Exercise sets[b]	5	268	0.294 ± 0.128	**2.29(0.02)***	--	--	--	--
Exercises (#)[b]	5	135	-0.037 ± 0.030	-1.21(0.23)	--	--	--	--
Exercise load rating	9	466	-0.00005 ± 0.0002	-0.25(0.80)	6	402	0.00006 ± 0.00016	0.41(0.68)
Baseline BMD	6	402	-1.889 ± 1.073	**-1.76(0.08)****	6	402	-0.010 ± 0.814	-0.01(0.99)
Body weight (Δ)	5	296	0.347 ± 0.200	**1.73(0.08)****	--	--	--	**--**
Strength – upper (Δ)	3	295	-1.605 ± 0.939	**-1.72(0.09)****	3	295	-0.82 ± 0.938	-0.88(0.40)
Strength – lower (Δ)	5	346	-0.156 ± 0.370	-0.42(0.67)	5	346	0.064 ± 0.323	0.20(0.84)

Notes: FN, femoral neck; LS, lumbar spine; BMD, bone mineral density; [a], limited to aerobic exercise; [b], resistance training only; Δ, changes in predictor variable; ES, effect size; #, number; Participants (#), number of exercise and control participants nested within ES's; $\beta_1 \pm$ SE, slope $\pm$ standard error; *Z(p)*, *z*-score and alpha value; *, statistically significant ($p \leq 0.05$); **, trend for statistical significance (>0.05 to $\leq$ 0.10); --, Insufficient data reported (< 3 ES's).
